# Adoption and Acceptability of a Fruit and Vegetable Subsidy in Low-Income Households in Urban Chile: The Bolsillo Saludable Pilot Feasibility Study

**DOI:** 10.1016/j.cdnut.2026.109394

**Published:** 2026-06-13

**Authors:** Isabel Pemjean, Daniela Montes de Oca, Jonathan Lara-Arévalo, Shu Wen Ng, Lindsey Smith Taillie, Camila Corvalán

**Affiliations:** 1 CIAPEC, Institute of Nutrition and Food Technology, University of Chile, Macul, Chile; 2 Institute of Economics, Pontifical Catholic University of Chile, Macul, Chile; 3 Department of Nutrition, Gillings School of Global Public Health, University of North Carolina at Chapel Hill, Chapel Hill, North Carolina, United States; 4 Carolina Population Center, Chapel Hill, NC, United States

**Keywords:** healthy food subsidy program, Latin America, adoption study, food security, food access

## Abstract

**Background:**

Low-quality diets disproportionally affect low-income populations, limiting access to healthy foods.

**Objectives:**

This feasibility study evaluated the adoption and acceptability of Bolsillo Saludable (BS), a smartphone app-based subsidy designed to incentivize fruit and vegetable (FV) purchases at Ferias Libres (open markets) in Chile.

**Methods:**

In an 8-wk pilot study, 30 low-income families received monthly benefits to purchase FV at a Feria Libre in Santiago, with outcomes assessed using a multimethod approach. Qualitative data from focus groups with beneficiaries (*n* = 15) and vendors (*n* = 6) explored acceptability and appropriateness. Quantitative data from the subsidy platform were used to assess adoption. Secondary outcomes (FV expenditure, purchases, dietary diversity) were assessed through app data records and pre–post surveys. Thematic and pre–post analyses were conducted.

**Results:**

The study demonstrated high adoption and acceptability of the program. All beneficiaries utilized their benefits, with at least 1 use each month. Qualitative results showed high satisfaction among beneficiaries and vendors, who appreciated the program’s focus on healthy food and the ease of the payment system. Participants perceived the restriction to FV and delivery through Ferias Libres as consistent with their purchasing practices, suggesting high appropriateness of the intervention. Over half of beneficiaries perceived positive changes in their family diet, including increased FV consumption and vendors noted increased sales of a wider FV variety. Quantitative results suggested a 38% increase in mean FV expenditure, with improvements in dietary diversity indicators. However, qualitative results identified challenges related to variety of products offered, participating vendors, and invoicing difficulties.

**Conclusions:**

This feasibility pilot demonstrated high adoption, acceptability, and appropriateness of the BS program with preliminary evidence of positive dietary changes among vulnerable populations. Addressing operational challenges, particularly limited vendor participation and invoicing barriers will be essential for scale-up. These findings provide key insights for scaling this intervention in Chile and adapting similar programs across similar settings.

This trial was registered at University of Chile Protocol Record 5128637 as NCT06865157; https://clinicaltrials.gov/study/NCT06865157

## Introduction

The high prevalence of low-quality diets, characterized by excessive consumption of unhealthy foods (e.g., high in sodium, sugars, saturated fats, and calories) and inadequate intake of fresh foods (including fruits, vegetables, legumes, and whole grains), poses a significant global public health concern. Research indicates that unhealthy diets are responsible for ∼20% of global deaths [1]. Moreover, the consumption of ultraprocessed foods (UPFs) has been linked to an increased risk of noncommunicable diseases and some cancers [2]. The growing availability, aggressive marketing, and affordability of UPFs have contributed to their increased consumption, displacing healthier food options [3]. The COVID-19 pandemic, recent conflicts, and extreme weather events have exacerbated the deterioration of diet quality and contributed to the rising costs of food [4,5]. Furthermore, unhealthy diets are a significant factor in health disparities, with evidence indicating that households of lower socioeconomic status (SES) tend to have poorer dietary quality compared with wealthier households [6].

Improving diet quality requires reducing unhealthy food consumption while increasing the intake of healthier options [7]. Many countries have implemented public health measures to reduce the consumption of unhealthy foods, including policies such as front-of-package (FOP) labels [8], taxes on foods high in nutrients of concern and sugar-sweetened beverages (SSB), and restricting unhealthy food availability in schools [9]. These measures have been shown to positively influence product reformulation [10] and consumption patterns [11].

Chile has been a leader in implementing comprehensive regulations to limit unhealthy food consumption, most notably through its Food Labeling and Advertising Law. This law mandates FOP labels for foods high in nutrients of concern, restricts marketing, and limits the availability of such foods in schools [9]. The country has also imposed small taxes on SSB. Although these measures have shown positive outcomes including reductions in purchases of foods high in sugars, sodium, and saturated fats [12], Chile continues to struggle with poor overall dietary quality, particularly due to low consumption of FV [13]. Affordability remains a significant barrier to healthy food consumption. A review found that low-income mothers viewed healthier foods as financially inaccessible [14], and in 2019, 3.8% of the population could not afford a healthy diet [15]. Additionally, the consumption of foods high in sugars, sodium, and saturated fats is more common in lower socioeconomic groups, whereas healthier food intake rises with SES [16]. This disparity is reflected in diet-related diseases such as hypertension, diabetes, and obesity, emphasizing the need to make healthy foods more accessible and affordable for lower-income individuals [17].

In Chile, healthy food subsidy programs (HFSP), which provide participants with cash to purchase healthy foods, emerge as a promising approach to improve dietary quality. Such programs could also positively impact traditional food purchases, shorten the agricultural supply chain, and build resilience among local farmers [18]. Across the country, Ferias Libres (i.e., open markets) provide ideal settings for healthy food subsidy initiatives as they offer fruits and vegetables (FV) at lower prices, are frequented by lower-income households [19], are largely supplied by regional producers, and have been recognized for promoting healthier food environments [20] while indirectly supporting local economies. However, Ferias Libres operate without standardized regulations. Each municipality defines their operational parameters, including permits, products sold, operating hours, minimum infrastructure, and size. Furthermore, Chile has 3 principal geographic zones (north, central, and south), characterized by distinct climatic and cultural conditions that may influence the functioning of Ferias Libres.

Mobile health (mHealth) and app-based interventions may facilitate benefit delivery, reduce administrative and participation barriers, improve transaction monitoring, and increase accessibility among low-income populations with growing smartphone access [21,22]. However, a recent global review highlighted the limited evidence available from low- and middle-income countries, particularly in real-world community food environments [22]. Evidence from Latin America is especially scarce, with little known about the feasibility, adoption, and implementation of smartphone-based HFSP in settings such as open-air markets.

As a result, significant knowledge gaps remain regarding how to effectively deliver and implement HFSP to maximize their reach, adoption, and potential impact, particularly among the most disadvantaged populations. Furthermore, it is essential to gather insights from potential beneficiaries and program implementers to ensure that these programs address the needs of the target population. To address this knowledge gap, the objective of this study was to evaluate the adoption and acceptability of a pilot feasibility study, the Bolsillo Saludable (BS) program, a smartphone app-based healthy food subsidy initiative, in the Chilean context. The program aimed to provide low-income families with an economic incentive to purchase FV at Ferias Libres. As secondary outcomes, we also assessed changes in FV expenditure, purchases, and dietary diversity. These results could guide and accelerate implementation of similar programs in other countries experiencing food insecurity.

## Methods

We employed a 2-stage process. First, we conducted a formative study to define the essential elements of a Chile-specific HFSP. Our methodological approach was grounded in evidence-based research methods to maximize the effectiveness of the intervention, which led to the design of the BS program—a mobile-based initiative aimed at promoting healthy eating habits among low-income families by incentivizing the purchase of FV at Ferias Libres. A community-based approach was utilized throughout the entire process. In the second stage, we conducted a pilot feasibility study that included implementation of the program and assessment of selected implementation outcomes.

### Background – formative research: developing the intervention

The formative stage comprised 4 components. The first component consisted of a narrative review synthesizing available evidence on HFSP worldwide (eligibility, design, implementation, and outcomes) [22]. The second component involved several formative activities conducted to enhance HFSP relevance and address implementation barriers:

*1*) A stakeholder matrix mapped relevant institutions for the BS program, categorized by power/influence and interest in Chile’s healthy food subsidy project. Semi-structured interviews with 14 key stakeholders (from governmental, nongovernmental, trade, civil, and academic sectors) explored their expectations, concerns, and proposals, aligning strategies with reality. Scalability, particularly regarding geographical differences, was addressed.
*2*) Go-along interviews (*n* = 191) [23], were conducted with *Feria* vendors (*n* = 52), regular *Feria* shoppers (*n* = 79), and nonshoppers (*n* = 60), using purposive sample to ensure regional (north, central, and south) and urban/rural diversity. Academic anthropologists explored motivations, consumption preferences, and purchasing behavior of FV through the interviews. Additionally, for *Feria* shoppers, nonshoppers, and vendors, topics included their perceptions of a potential subsidy, including preferred delivery mechanisms and participation barriers.
*3*) Academic anthropologists conducted field observations of the 18 Ferias Libres to understand their functioning, product range, internet availability, and purchasing dynamics, all key considerations for implementing a healthy food subsidy.

The third component involved estimating the benefit amount per eligible person based on the difference between the FV components of the Chilean Basic Food Basket (BFB) and a Quality Food Basket (QFB). The QFB was designed based on healthy diet guidelines, adjusting the proportions of food items in the BFB to healthier proportions and eliminating certain unhealthy items (e.g., confectioneries, SSB, and similar products) [24]. We considered the prices faced by the 20% of families with the lowest income in Chile (based on the Household Budget Survey IX 2021–2022) [25] and the inflationary pressures of the last 5 y, to estimate the difference between the FV products in the BFB and the QFB in 2024. The result was Chilean peso (CLP) 16,000 (∼USD 17) per month.

**FIGURE 1:**
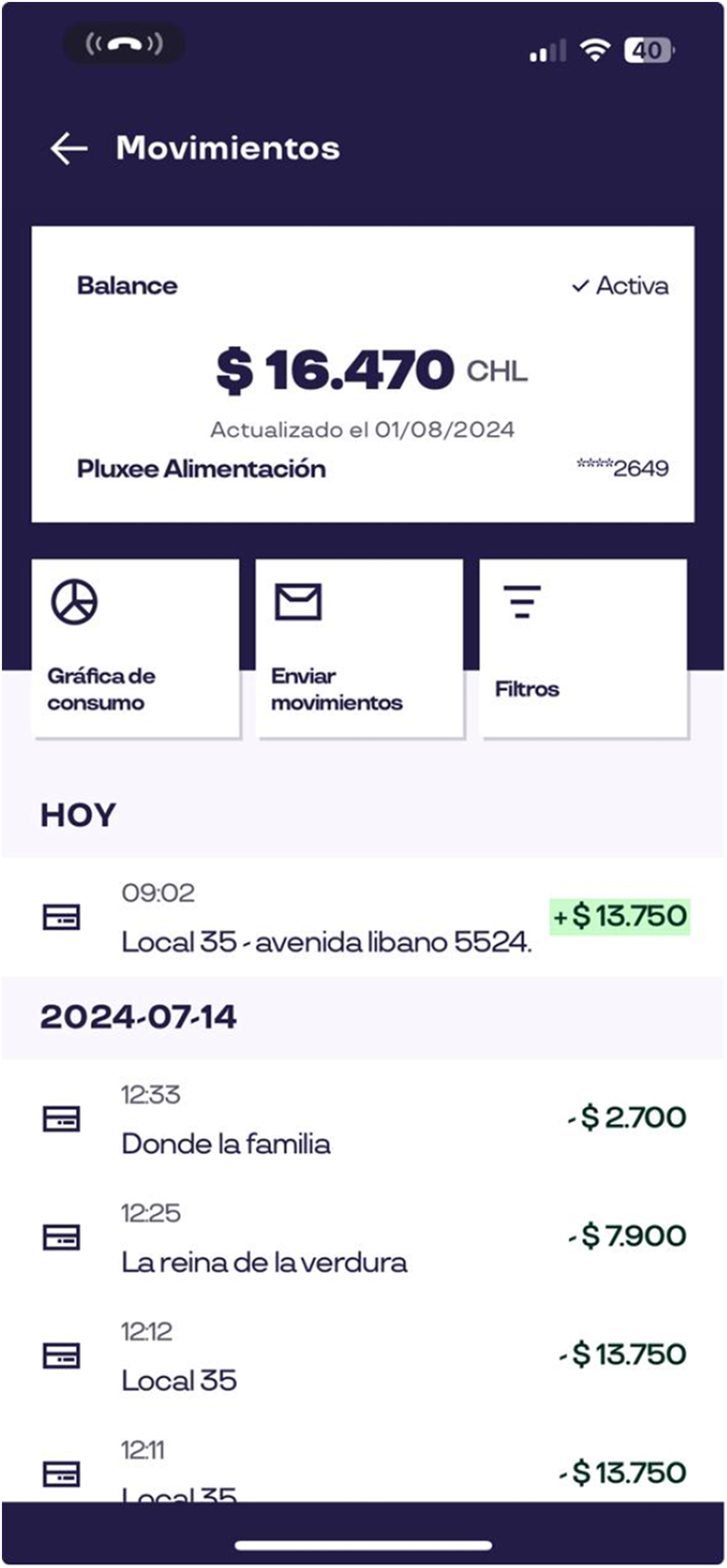
Mobile app interface.

Finally, the fourth component involved identifying and developing a delivery system of the economic incentive. The information obtained in the previous components (the narrative review and interviews with stakeholders, *Feria* shoppers, nonshoppers, and vendors) informed the selection of a mobile application (Figure 1) as the platform for the delivery and use of the BS program. This decision was supported by the widespread daily use of smartphones in Chile (94.9% of the urban population and 94.2% of the rural population) [26], indicating broad feasibility of an app-based delivery mechanism across population groups.

### Adoption and acceptability study: implementing and evaluating the intervention

Consistent with Proctor et al.’s implementation outcomes framework [27], this feasibility pilot study was designed to formally evaluate 2 primary implementation outcomes: adoption and acceptability. In addition, appropriateness—the perceived fit, relevance, or compatibility of the intervention for participants and the implementation setting—was explored as an emergent outcome through qualitative data, given its conceptual proximity to acceptability and its relevance for understanding participant fit with the intervention. Feasibility and sustainability were not formally operationalized as primary outcomes given the exploratory nature of the pilot; however, implementation challenges identified during the study are discussed in relation to these outcomes to inform future scale-up. Outcomes such as fidelity, penetration, and cost were beyond the scope of this pilot and represent priority areas for evaluation in future, larger-scale studies.

#### Ethical considerations

This study was conducted in accordance with the Declaration of Helsinki, and the Ethics Committee of the Faculty of Medicine (University of Chile) approved the protocol and informed consent. All participants (beneficiaries and vendors) signed the informed consent form.

#### Study design

**FIGURE 2:**
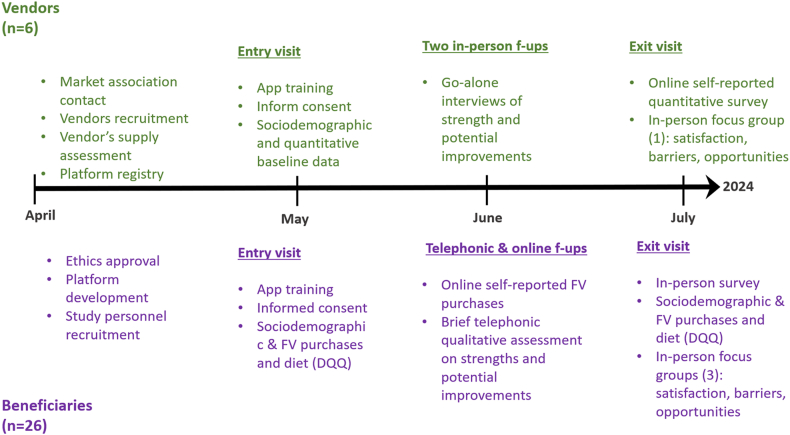
Study design.

We conducted a single-arm feasibility and acceptability pilot with pre–post evaluation. The BS pilot was implemented over an 8-wk period during June–August 2024 combining multiple method’s assessment (Figure 2). Consistent with Proctor et al.’s implementation outcomes framework [27], the study focused on 2 implementation outcomes: adoption and acceptability. The qualitative assessment included focus groups conducted among all vendors and a subset of beneficiaries, and the quantitative assessment included platform-based indicators of subsidy use and pre–post measures among participating beneficiaries. In addition, implementation experiences and challenges identified during the pilot informed the discussion of feasibility and sustainability considerations relevant to future scale-up.

#### The intervention

The Bolsillo Saludable program provided a monthly electronic credit of 16,000 CLP (∼17 USD) per eligible household member, including children under 18 y, students under 25 y, and individuals with disabilities, to purchase FV at a Feria Libre (not all the household’s members were eligible to receive the monthly credit). The recipient of the household’s total Bolsillo Saludable allocation (the beneficiary) was the household’s primary food purchaser (identified during recruitment).

The program operated through a mobile app with 2 interfaces: one for the Bolsillo Saludable beneficiaries and another for FV vendors. Both parts were required to download the app on their mobile devices and create a personal account. A pre-existing private mobile application, currently used to deliver university food benefits within a governmental program, was adapted for the purposes of the pilot.

The total Bosillo Saludable allocation of the household was monthly loaded on the beneficiary personal app account that allowed users to view available funds, make purchases from authorized vendors at the *Feria Libre*, and review past purchases.

At the *Feria Libre*, FV vendors registered for the Bolsillo Saludable program, and each was provided with a distinctive program identification sign featuring a unique code. To make a purchase, the beneficiary had to enter the stall’s code into the app, input the amount to be paid, and authorize it with their password. The app automatically generated a purchase receipt on the beneficiary’s interface and deducted the amount from their balance.

The following restrictions were established for the use of the Bolsillo Saludable on the app:

*1*) The Bolsillo Salusable could only be used through the app to purchase FV to registered stalls from the *Feria Libre.*
*2*) It was impossible to withdraw the funds as cash.
*3*) Any FV could be purchased on the registered stall from the *Feria Libre.*
*4*) The funds could be rolled over from the 1st to the 2nd mo, allowing beneficiaries to accumulate unused funds.

There were no other limitations on the Bolsillo Saludable use, such as the amount of money spent per stall or minimum/maximum visits to the *Feria Libre*.

As for the vendors, every time a purchase was made, they received a payment receipt on their app interface. Their interface kept a record of all their sales along with the necessary information to invoice them. Considering the vendors’ need for readily available funds, a 2-d rapid payment system (bank transfer) was implemented. No additional criteria or administrative requirements were imposed; vendors only needed to generate the invoice to receive payment.

#### Setting

Because the study focuses on low-income households, we selected a low-income population in urban Santiago (the largest city in Chile) for the pilot. Specifically, we selected the Villa La Fundación and Villa Las Cumbres neighborhoods, whose population is among the 40% most vulnerable population in Chile [28]. This area is also within a 10-min walk from the *Feria Libre* Juan Pinto Durán (JPD), which operates on Thursdays and Sundays from 08:00 to 15:00. The Feria JPD has more than 100 stalls. Over half of them sell FV, as well as eggs, cheese, legumes, fish, and seafood. The other half sell clothing, plants, electronics, kitchenware, and packaged foods. Packaged items include products with and without FOP labels (e.g., pasta, rice, dairy, cookies, and snacks). Before the intervention, only 10% of the stalls had signs announcing availability of electronic payments, although others offered this option without signage.

#### Beneficiaries sample

Thirty households received the Bolsillo Saludable intervention for 2 consecutive months. The eligibility criteria was that the households had participated in the *Bolsillo Familiar Electrónico* (BFE) in 2023–2024. The BFE is a government subsidy designed to mitigate the impact of rising food prices during the COVID-19 pandemic. BFE beneficiaries were selected based on other governmental programs such as *Subsidio Familiar*, *Asignación Familiar o Maternal*, and *Subsistema de Seguridades y Oportunidades*. These programs have their own specific inclusion criteria, but they all target low-income families with dependents, whether they are children, adolescents, or children with disabilities. To be eligible as the BS beneficiary (i.e., the recipient of the total Boslsillo Saludable allocation for the household), the following criteria had to be met: *1*) be at least 18 y old (legal age), *2*) be a parent or guardian of a child aged 0–5 y, and *3*) be responsible for the household’s food purchases. Vendors at the *Feria Libre* JPD were excluded. Although the intervention relied on a smartphone application, access to a smartphone or digital literacy skills were not included as formal eligibility criteria. This decision was informed by the high penetration of smartphones, widespread internet connectivity, and the increasing use of mobile applications for everyday activities in urban Chile, particularly following the COVID-19 pandemic [26] and the intention to evaluate the feasibility of implementing this technology under real-world conditions. Beneficiaries were recruited from neighborhood councils to facilitate community-based outreach to potentially eligible households. Participants were screened for eligibility until reaching the 30 participating households.

#### Vendors sample

To be eligible, the stalls at the Feria JPD had to *1*) be exclusive FV vendors, and *2*) agree to sign a contract with the app company due to financial transactions, to ensure payment for transactions, and to grant permission to access and share transaction data. The invitation was done through the current vendors’ association leader. Thirty vendors expressed oral interest in participating, and 8 of them attended the project’s initial meeting (vendors Event 1). Of the 8 vendors who signed the contract, 6 ultimately participated in the Bolsillo Saludable program. Two other vendors did not participate due to concerns about the payment process.

#### Study implementation

***Early stage:*** Beneficiaries and vendors were trained on the mobile app. Vendors also received information on the benefit payment system, including invoice issuance and rapid 2-business-day payment processing (bank transfer).

***Follow-up:*** To ensure successful implementation, regular follow-up sessions were conducted to monitor progress, address challenges, and provide targeted support. A mystery user profile was included as a beneficiary, to identify key issues. This mystery user was unknown by the app company, beneficiaries, and vendors. We also conducted go-along interviews [29] with vendors and brief phone qualitative assessment with beneficiaries to address difficulties, strengths, and improvements of the Bolsillo Saludable program.

### Implementation outcomes

*1*) **Adoption (beneficiaries):** We assessed the adoption of the intervention through: (A) the percentage of the monetary subsidy that was spent at the end of the pilot based on the records from the subsidy delivery platform, and (B) the percentage of beneficiaries that used the monetary subsidy at least once per month, based on the records from the subsidy delivery platform.
*2*) **Acceptability (beneficiaries and vendors) and appropriateness:** The acceptability of the intervention was assessed through: perceived utility of the subsidy program (A), perceived utility of the subsidy platform (B), self-reported satisfaction with the subsidy program (C), and self-reported satisfaction with the subsidy platform (D). Outcomes A and B were evaluated via online self-reported exit surveys, while outcomes C and D were explored through exit focus groups.

Beneficiaries’ online self-reported exit survey included the evaluation of the Bolsillo Saludable utility (outcome A) on affordability, quantity, and quality of FV purchases, and on ease of use of the platform (outcome B). Responses were rated on a 5-point Likert scale and categorized as follows: “Agree” (1: strongly agree/2: agree), “Neither agree nor disagree” (3), and “Disagree” (4: disagree/5: strongly disagree).

Vendors’ online self-reported survey included the evaluation of the Bolsillo Saludable utility (outcome A) by the perceived value of participation, considering time spent on training, troubleshooting, and the program’s benefits to their work and, the ease of use of the platform (outcome B), rated on a 5-point Likert scale. Responses were rated on a 5-point Likert scale and categorized as follows: “Agree” (1: strongly agree/2: agree), “Neither agree nor disagree” (3), and “Disagree” (4: disagree/5: strongly disagree).

Three exit focus groups were conducted with beneficiaries (*n* = 15) and one with vendors (*n* = 6) at Centro de Investigación en Ambientes Alimentarios y Prevención de Enfermedades Crónicas asociadas a la alimentación- Instituto de Nutrición y Tecnología de los Alimentos (CIAPEC-INTA), all audio-recorded complemented the quantitative assessments.

Appropiateness was explored qualitatively through participants’ and vendors’ perceptions regarding the suitability of restricting the subsidy to FV and delivering it through Ferias Libres.

*3*) **Feasibility and sustainability of the program:** We explored implementation challenges among beneficiaries and vendors to better understand factors affecting the feasibility and potential sustainability of the intervention. These challenges were assessed through exit focus groups conducted with beneficiaries (*n* = 15) and vendors (*n* = 6) at the CIAPEC-INTA facilities. All sessions were audio-recorded.

### Other outcomes

1) **FV Expenditure (beneficiaries):** The amount of financial resources allocated by beneficiaries to purchasing FV was assessed by (A) the self-reported monthly expenditure on all fruits purchased from the *Feria Libre*, (B) the self-reported monthly expenditure on all vegetables purchased from the *Feria Libre*, and, (C) the self-reported monthly expenditure on all FV combined, purchased from the *Feria Libre.* The data for these 3 outcomes were based on a survey at baseline and at the end of the study. Expenditures were calculated based on the reported purchases and the prices of these products during the reference week obtained from national statics [30].
2) **FV purchases (beneficiaries):** Quantity of FV acquired by beneficiaries were measured through (A) the self-reported monthly fruit purchases (in kilograms) at the *Feria Libre*, (B) the self-reported monthly vegetable purchases (in kilograms) at the *Feria Libre* and, (C) the self-reported monthly vegetable purchases (in units) at the *Feria Libre.* The list consists of 11 fruits and 12 vegetables most frequently reported by schoolchildren and pregnant women from cohorts conducted by CIAPEC-INTA [31]. The data for these 3 outcomes were based on a survey at baseline and at the end of the study.
3) **Dietary diversity (beneficiaries):** measured using the Dietary Quality Questionnaire (DQQ). The DQQ is an internationally standardized dietary assessment tool that allows a feasible short dietary measurement (5 min) and provides a full description of dietary data [32]. It consists of yes/no questions about foods or beverages consumed the previous day using 29 food groups that have been adapted to the Chilean context. Detailed information on the DQQ methodology, country-specific adaptations, and indicator construction is publicly available through the Diet Quality Questionnaire platform (https://www.dietquality.org/), which includes the Chilean adaptation used in this study. Four DQQ indicators were assessed at baseline and exit: the NCD-Protect score (0-9), reflecting consumption of health-promoting foods; the NCD-Risk score (0-9=, reflecting consumption of foods to limit or avoid; the Global Dietary Recommendations score (GDR, 0–18), a composite measure of overall diet quality (GDR = NCD-Protect − NCD-Risk + 9); and the Food Group Diversity Score (FGDS, 0–10), a proxy for micronutrient adequacy. Higher GDR, NCD-Protect, and FGDS scores indicate better diet quality; a higher NCD-Risk score indicates poorer quality.

A survey, including FV purchases and the DQQ were conducted at the baseline and at the end of the study (0–2 mo). The initial survey about FV purchases was self-administered online, whereas the initial DQQ was conducted in person. The subsequent surveys were administered in person by the research team.

### Beneficiaries baseline demographic and socioeconomic measures

At baseline and exit, beneficiaries were asked about age, sex, education, head of the household, household size, work, and whether the household could afford to pay their bills and buy fruits, vegetables, and other products that the family desired.

### Vendors baseline demographic and socioeconomic measures

At baseline, vendors were asked about age, sex, types of food sold, electronic sales before the Bolsillo Saludable program, if they had concerns about food supply shortages or about quality of food for sale.

### Qualitative analyses

A social anthropologist conducted a thematic analysis of the data using MAXQDA software v.2024. This analysis employed a deductive coding approach, informed by the study objectives related to program acceptability. Specifically, the analysis focused on examining beneficiaries’ satisfaction with the Bolsillo Saludable program, mobile application, and their perceptions about implementation challenges. Thematic saturation was reached, as no new themes emerged during the final stages of analysis. Triangulation across different participant groups and qualitative data sources was used to strengthen interpretation of the findings.

### Statistical analyses

We conducted an exploratory pre–post analysis to descriptively assess potential changes during the intervention rather than formally evaluate intervention efficacy, given the nature of the study and limited sample size. The following variables were analyzed: total expenditure on FV, fruit expenditure, vegetable expenditure, kilograms of fruits purchased, kilograms and units of vegetables purchased, and consumption scores such as the GDR score, NCD-Protect, NCD-Risk, and FGDS. Exploratory paired *t*-tests were conducted to estimate mean pre–post differences, corresponding 95% confidence intervals, and 2-sided *P* values among participants with complete pre- and post-intervention data. These parametric tests were used to assess whether the mean pre–post difference within participants was equal to zero. Analyses were restricted to complete cases only. Data on these variables were available at both baseline and pilot exit for 26 beneficiaries. All analyses were conducted using STATA v16 (StataCorp LLC, College Station, TX, USA).

## Results

### The beneficiaries and vendors

All beneficiaries were women, i.e., women were the food purchasers in all households. Almost all (82.4%) were under the age of 40 y. At the onset of the pilot, over 60% were employed and held the position of head of household. Generally, the households were characterized by a large size (68% >3 members) and a precarious economic situation, with over half of them experiencing difficulties in paying bills and reducing expenses. More than half of the households (57.7%) had 1 to 2 eligible members for the Bolsillo Saludable (Table 1). Although it was not an inclusion criterion, all the beneficiaries already shopped at a *Feria Libre*, even if it was not the *Feria Libre* JPD.

**TABLE 1:** Sociodemographic characteristics of the beneficiaries

Characteristics	Percentage (*n* = 26)
Age (y)	
21–30	46.2
31–40	46.2
41–45	7.6
Employment status (% of beneficiaries that work full time)	
Baseline	50.0
Exit	46.2
Employment status (% of beneficiaries that work part time)	
Baseline	11.5
Exit	11.5
Sex head of the household, woman	64.0
Household size	
1–3	32.0
>3	68.0
Financial situation (% of beneficiaries that struggles to pay and covers bills by cutting back)	
Baseline	57.7
Exit	69.2
Number of eligible household member	
1–2	57.7
3–4	42.3

During the pilot, 6 vendors participated in the Bolsilo Saludable program. Three of them were over 50 y old and 4 were women. All vendors were stall owners, and sold either fruit or vegetables. Notably, all vendors had previously accepted electronic payments, which accounted for approximately half of their total sales in 5 out of the 6 cases (Table 2). The benefit attracted larger Feria vendors with a wider range of products and higher prices.

**TABLE 2:** Sociodemographic characteristics of the vendors

Characteristics	Percentage (*n* = 6)
Age (y)	
21–30	16.7
31–40	33.3
>50	50.0
Sex, woman	66.7
Type of food sold	
Fruits	50.0
Vegetables	50.0
Nuts, cheese, fish, meat, or pulses	0
Electronic sales before Bolsillo Saludable program	
Almost nothing	16.7
Half of total sales	83.3
Having concerns about food supply shortages	0
Concerns about quality of food for sale	
Often	33.3
Never	66.7
Oversupply, rarely	66.7

### Adoption results

**FIGURE 3:**
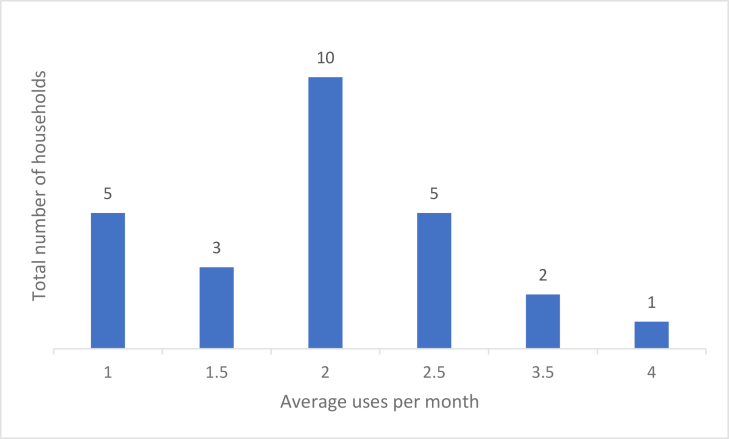
Number of beneficiaries by Bolsillo Saludable average use per month.

A total of 97.9% of the monetary subsidy was spent by the end of the pilot. According to the number of eligible members in each household, the beneficiaries received on average 38,153 CLP (∼40 USD) monthly. All of the beneficiaries used on average the Bolsillo Saludable at least once a month and 69.2% of the beneficiaries shopped at the Feria on average 2 or more times a month (Figure 3). Beneficiaries preferred to shop on Sundays rather than Thursdays (Supplemental Figure 1). Taking into account all transactions carried out with the Bolsillo Saludable, the distribution of beneficiaries and money spent were not uniform across the *Feria Libre* vendors (Supplemental Figure 2).

### Acceptability and appropiateness results

#### Beneficiaries’ acceptability of the program

**FIGURE 4:**
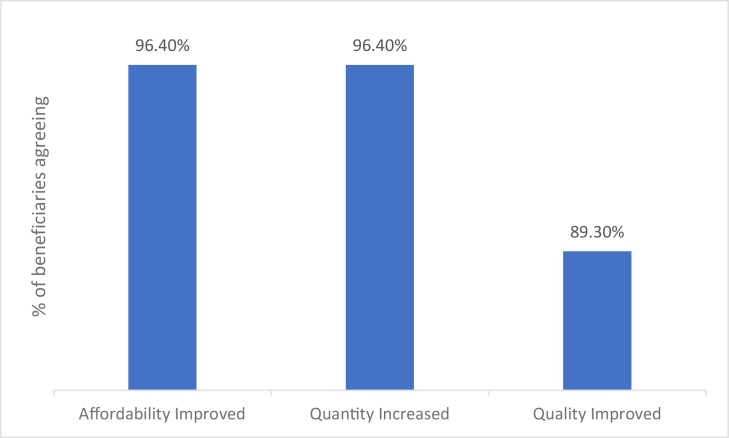
Beneficiaries’ agreement on the perceived utility of the Bolsillo Saludable program on Fruit and Vegetable affordability, quantity, and quality (*n* = 26).

A total of 96.4% of beneficiaries agreed that the Bolsillo Saludable program was useful in enhancing their ability to afford FV and increasing the quantity of FV they purchased. Additionally, 89.3% reported that the program enabled them to improve the quality of FV they acquired (Figure 4).

**FIGURE 5:**
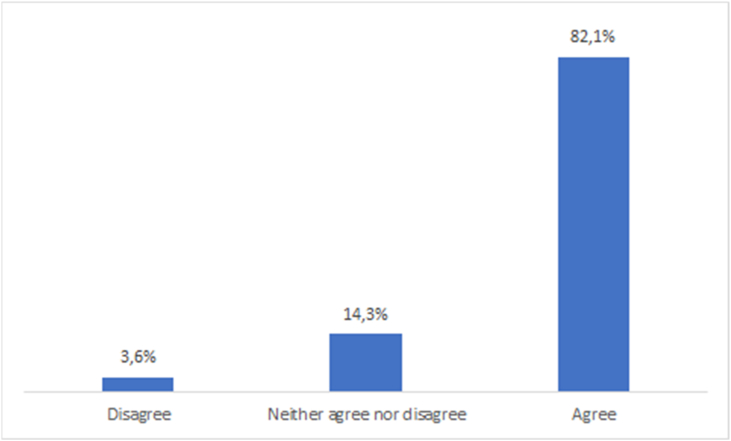
Beneficiaries (*n* = 26) perception of the mobile application utility. Was it easy to use the mobile application?

A total of 82.1% of beneficiaries reported that the mobile application was easy to use (Figure 5). Qualitative findings were consistent with the quantitative acceptability results and provided additional insight into the beneficiaries’ experiences with the Bolsillo Saludable program, including perceived benefits, usability of the mobile application, perceived dietary changes, and implementation challenges (Table 3).

**TABLE 3:** Beneficiaries’ qualitative perceptions of the Bolsillo Saludable program: themes, representative quotes, and implementation implications

Theme	Subtheme	Representative quote	Implementation implication
Perceived economic benefits	Improved affordability of FV	“It [Bolsillo Saludable] was very useful for me. I took full advantage of it.” (Beneficiary FG2)	Subsidies may improve perceived economic access to healthy foods among low-income households.
Acceptability of healthy food restrictions	Positive perceptions of restricting subsidy to FV and Ferias Libres	“That way one prioritizes fruits and vegetables.” (Beneficiary FG1)	Restricting subsidies to healthy foods was acceptable and perceived as supportive of healthier purchasing behaviors.
Acceptability of digital delivery system	Ease of use of the mobile app	“Using the app was straightforward…” (Beneficiary FG1)	Smartphone-based delivery systems may be feasible in urban settings with high digital connectivity.
Perceived dietary changes	Increased FV consumption and replacement of unhealthy snacks	“My son ate more fruit.” (Beneficiary FG2)	Participants perceived positive changes in household dietary practices during the pilot.

##### Beneficiaries’ perceptions of economic and dietary benefits

The qualitative results indicated that beneficiaries were highly satisfied with the Bolsillo Saludable program, mainly for economic reasons.

“*I go to the fair with the money the children's father gives me, and he doesn't have work every week. If there is no work, there is no Feria at the weekend. So, it [Bolsillo Saludable] was very useful for me. I took full advantage of it*” (Beneficiary FG2).

##### Acceptability of healthy food restrictions

It was appreciated that the Bolsillo Saludable program was limited to healthy food (FV). Beneficiaries stated that if the subsidy was freely available, it would be spent mainly in supermarkets and on lower quality food.

“*If it [Bolsillo Saludable] were free, people would have spent more in supermarkets than in Ferias. As it is, it's fine, that way one prioritizes fruits and vegetables*” (Beneficiary FG1).

“*It is good that they put clauses because that way they are going to make sure that the people who had the benefit spend it well. If they don't put a clause, you can spend it on X unhealthy things*” (Beneficiary FG2).

##### Acceptability of the mobile application

Additionally, beneficiaries found the payment application effective once they mastered its use.

“*Using the app was straightforward; I just entered the code from the stall poster and input my spending amount – it was incredibly user-friendly*” (Beneficiary FG1).

##### Perceived dietary and behavioral changes

Beneficiaries perceived that the Bolsillo Saludable program was useful in changing the habits of their families. More than half of the beneficiaries reported a positive difference in their family’s diet as a result of the Bolsillo Saludable:

Some beneficiaries reported replacing unhealthy snacks with fruit:

“*Even though there was no possibility to buy snacks for schools, because you buy at the Feria, you still send fruit. It is still a luxury saving*” (Beneficiary FG3).

Increased fruit consumption among children was noted:

“*My son ate more fruit. He saw the table full of fruit and wanted to eat all the time*” (Beneficiary FG2).

Increased vegetables consumption in adults was also reported:

“*In my house they don't eat much salad but when I came to the Feria I bought a lot of salad so they started to eat more, more types of salad*” (Beneficiary FG1).

#### Vendors’ acceptability of the program

**FIGURE 6:**
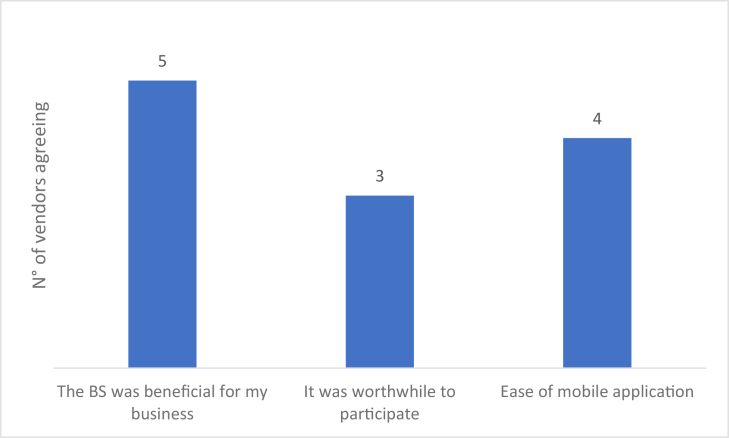
Vendors’ acceptability of the Bolsillo Saludable program (*n* = 6).

Among the 6 vendors surveyed, 5 reported that the subsidy program was beneficial to their business. Three vendors agreed that participation in the program was worthwhile, whereas 1 vendor disagreed and 4 reported that the mobile application was easy to use (Figure 6).

Qualitative findings were consistent with the quantitative acceptability results and provided additional insight into the vendors’ experiences with the BS program (Table 4).

**TABLE 4:** Vendors’ qualitative perceptions of the Bolsillo Saludable program: themes, representative quotes, and implementation implications

Theme	Subtheme	Representative quote	Implementation implication
Perceived economic benefits	Economic benefits for vendors	“It's a win–win situation.” (Vendor 4)	Vendor participation may support both food access and local economic activity.
Perceptions of healthy food focus	Positive views of restricting subsidy to healthy foods	“This was food, and healthy food.” (Vendor 1)	Vendors also perceived value in maintaining healthy food restrictions.
Acceptability of digital delivery system	Positive perceptions of the payment system	“The purchasing system was remarkably efficient.” (Vendor 2)	Digital payment systems may facilitate implementation once users become familiar with the platform.
Perceived implementation opportunities	Greater FV variety and strengthened vendor-customer relationships	“It was a more familiar relationship.” (Vendor 2)	Programs implemented through Ferias Libres may strengthen social interactions and diversify FV purchases.

##### Vendors’ perceptions of economic benefits

The qualitative results indicated that vendors were also highly satisfied with the Bolsillo Saludable program, mainly for economic reasons.

“*I understand that there are people who need it [Bolsillo Saludable]. It's a win–win situation*” (Vendor 4).

“*On a personal level, it helped me a lot because these months are really tough for us, and honestly, that assistance [the sales increases thanks to the Bolsillo Saludable] was really great*" (Vendor 1).

##### Acceptability of healthy food restrictions

It was appreciated that the Bolsillo Saludable program was limited to healthy food (FV).

“*People have money specifically allocated for buying fruits and vegetables, and obviously, when we're talking about health, it's a really great project*” (Vendor 5).

“*Because sometimes when people get some vouchers they spend them on other things that are not really necessary. But this was food, and healthy food*” (Vendor 1).

##### Acceptability of the mobile application

Vendors found the payment application effective once they mastered its use.

“*The purchasing system was remarkably efficient, with no issues whatsoever*” (Vendor 2).

##### Perceived dietary and behavioral changes

Vendors perceived that customers were buying a wider variety of FV, including more expensive products in larger quantities, such as avocados. In addition, although less frequently mentioned, vendors mentioned an increase in social capital in exchanges with beneficiaries.

*“With the beneficiaries who bought, it was a more familiar relationship*” (Vendor 2).

#### Appropriateness

Beyond acceptability, the findings also suggest a high degree of appropriateness of the intervention. Participants perceived the restriction of the subsidy to FV as consistent with the program’s objective of promoting healthier diets, rather than as an undesirable limitation. Similarly, the use of Ferias Libres as the exclusive retail venue aligned well with participants’ existing food purchasing practices and was not perceived as a barrier to participation. Beneficiaries also reported incorporating the subsidy into their routine shopping activities, suggesting that the intervention was compatible with household food acquisition practices and local food environments.

### Feasibility and sustainability

#### Beneficiaries

The primary concern expressed by the beneficiaries was the limited availability of stalls at the Feria that accepted the Bolsillo Saludable. This resulted in perceived restrictions on the program, specifically: limited variety of FV available at vendor stalls and constraints on beneficiaries’ usual coping strategies, such as comparing prices across different stalls and distributing purchases across multiple Ferias.

“*I was counting on variety. I went with the expectation that I would take some of this, some of that. And I arrived at the Feria and there were limited stalls*” (Beneficiary FG1).

“*At that time, I went to [another] Feria that is very cheap, so I saw the prices and I thought, no, with all this money I would have bought more. So, what I bought here, I could buy twice as much there*” (Beneficiary FG3).

To overcome these problems, the beneficiaries suggested 3 key areas for improvement: *1*) expanded stalls and product options at Ferias including legumes, eggs and fish, *2*) expanded food outlets options to include larger food markets and, *3*) clear information on enrolled vendors and locations through the app, including maps, provided.

#### Vendors

Vendors faced some challenges during implementation, mainly with the payment system, as most had no prior experience with invoicing, making this process particularly challenging. However, the app company provided support through visits and tutorials.

“*It’s that if we had known from the beginning about internal taxes..., it would have been easier*” (Vendor 6).

To overcome these issues, vendors suggested improving initial processes with a focus on resolving payment problems and providing training.

### Other outcomes

In Table 5 we present results on FV expenditure, FV purchases, and dietary diversity, including pre–post differences, 95% confidence intervals, and *P* values from exploratory paired *t*-tests of mean differences to compare the observables indicators of the beneficiaries before and after the intervention. There were observable changes in beneficiaries’ purchasing and dietary indicators during the pilot, although the exploratory nature of the study, small sample size, short intervention duration, and absence of a control group limit strong conclusions regarding these findings.

**TABLE 5:** Means and difference of FV expenditure, purchases and dietary diversity indicators at baseline and exit of the Bolsillo Saludable intervention (*n* = 26)

	Mean pre-Bolsillo Saludable [95%CI^1^]	Mean post-Bolsillo Saludable [95%CI]	Difference pre–post^2^ [95%CI]	*P* value
Total expenditure on FV^3^ (CLP^4^)	15,895.89	21,945.33	6049.43	0.006
	[11,797.19, 19,994.60]	[18,072.54, 25,818.12]	[1881.60, 10,217.26]	
Expenditure on fruits (CLP)	6184.22	8396.25	2212.03	0.111
	[3850.83, 8517.61]	[6312.56, 10,479.94]	[−546.05, 4970.11]	
Expenditure on vegetables (CLP)	9711.67	13549.08	3837.40	0.001
	[7189.11, 12234.23]	[10,849.14, 16,249.01]	[1778.83, 5895.98]	
Purchases kilos of fruits	5.38	8.29	2.90	0.008
	[3.79, 6.98]	[6.46, 10.11]	[0.84, 4.97]	
Purchases kilos of vegetables	4.71	5.27	0.56	0.395
	[3.39, 6.04]	[4.08, 6.46]	[−0.77, 1.88]	
Purchases units of vegetables	4.65	6.04	1.38	0.054
	[3.31, 6.00]	[4.35, 7.73]	[−0.02, 2.79]	
GDR score^5^	9.81	10.46	0.65	0.177
	[8.96, 10.66]	[9.38, 11.54]	[−0.32, 1.62]	
NCD – Protect score^6^	3.15	4.00	0.85	0.003
	[2.67, 3.63]	[3.38, 4.62]	[0.33, 1.37]	
NCD – Risk score^7^	2.35	2.54	0.19	0.625
	[1.72, 2.97]	[1.75, 3.32]	[−0.61, 0.99]	
Food Group Diversity Score^8^	5.69	6.15	0.46	0.083
	[5.20, 6.19]	[5.65, 6.66]	[−0.06, 0.99]	

1 95% confidence intervals.

2 Differences are calculated as post-intervention minus pre-intervention values.

3 Fruits and vegetables.

4 Chilean pesos (1 USD = 951 CLP in June 28, 2024).

5 The GDR-score values range from a minimum of 5 to a maximum of 15.

6 The NCD-protect score values range from a minimum of 1 to a maximum of 8.

7 The NCD-risk score values range from a minimum of 0 to a maximum of 6.

8 The Food Group Diversity Score values range from a minimum of 3 to a maximum of 8.

Considering the 8 fruits and 11 vegetables included in the survey for which both consumption and price data were available, the mean increase in total FV expenditure relative to baseline was $6049 CLP/wk or 7.45 USD (38%, CI: 1881.60, 10217.26; *P* value: 0.006), despite a subsidy of at least $16,000 CLP. This increase appeared to reflect higher expenditure on both FV. In more detail, out of the 8 fruits, only lemons and strawberries showed lower average expenditure at follow-up, which may have been influence by seasonality and winter months. Despite the decrease in expenditure, the average of kilograms of lemons purchased was slightly higher (Supplemental Figure 3).

The average expenditure of each vegetable increased, whereas the average purchase (in kilograms or units) of lettuce was the only one that showed a slight nonsignificant decrease. Purchase of the rest of the vegetables and fruits were generally sustained or increased during the pilot period (data not shown).

Results about dietary diversity suggested trends toward higher adherence to global dietary recommendations at the end of the pilot (GDR score pre–post difference = 0.65; CI: −0.32, 1.62; *P* value: 0.177), including dietary factors considered protective against noncommunicable diseases (NCD-Protect score pre–post differences = 0.85; CI: 0.33, 1.37; *P* value: 0.003).

The FGDS also increased (pre–post difference of 0.46; CI: –0.06, 0.99; *P* value: 0.083), suggesting a trend toward greater dietary diversity among beneficiaries’ households. These findings should be interpreted cautiously, as expenditure and purchase measures relied partly on self-reported data and may also have been influenced by seasonal variability in FV prices and availability.

## Discussion

This pilot feasibility study evaluated the adoption and acceptability of Bolsillo Saludable, a smartphone app-based healthy food subsidy program designed to support FV purchases at Ferias Libres among low-income households in urban Chile. Guided by Proctor et al.’s implementation outcomes framework [27], the study examined adoption and acceptability of the intervention, while implementation challenges provided insight into feasibility and sustainability considerations for future scale-up. From an implementation science perspective, the findings suggest that the BS program achieved favorable implementation outcomes across multiple domains. High levels of subsidy utilization and regular participation indicate strong adoption, while positive perceptions among beneficiaries and vendors reflect high acceptability. Findings also suggest a high degree of appropriateness, as key program features—including the restriction of subsidies to FV and delivery through *Ferias Libres*—aligned well with participants’ needs, food purchasing practices, and expectations regarding healthy eating. At the same time, operational challenges identified during implementation highlight important feasibility considerations. Finally, the program’s reliance on existing social protection infrastructure and digital delivery systems provides insights into its potential sustainability and scalability.

The use of *Ferias Libres* as the exclusive retail venue appeared to contribute to several implementation outcomes. The strong territorial presence of *Ferias Libres* and their integration into participants’ existing purchasing routines likely supported both appropriateness and feasibility, while positive perceptions among beneficiaries and vendors suggest that this implementation was acceptable to key stakeholders. *Ferias Libres* are particularly prevalent in lower-income urban sectors in Chile [33]—attracting ∼2.5 million visitors in Santiago alone on weekends [34]—and are part of traditional food markets across Latin America that connect small-scale producers directly with urban consumers [35], suggesting potential applicability of this implementation strategy in other countries where traditional food markets play a central role in food access. Unlike large-scale programs where access to participating outlets has been identified as a challenge [36], the *Feria Libre* venue was not perceived as a barrier to participation, likely due to this strong territorial presence. The subsidy restriction to FV purchases at *Ferias Libres* was well received by both beneficiaries and vendors, and may have contributed to prioritizing healthy food purchases while supporting local traders, consistent with findings from pilots in England [37], France [38], and Ethiopia [39].

Interestingly, while previous evaluations suggest that participants typically favor subsidies with fewer restrictions for greater flexibility and autonomy [40], participants in our study appeared to value the focus on healthy foods and food environments. This finding may reflect an awareness among participants of the importance of consuming healthy foods and the necessity of navigating healthier food environments to make such choices. Chile has implemented a comprehensive set of policies to discourage the consumption of unhealthy foods, most prominently through the Food Labeling and Advertising Law. Although these policies have not fully addressed persistent inequalities in diet quality and affordability, sustained exposure to this regulatory environment may have contributed to shaping public understanding of healthy and unhealthy foods. Although our study did not aim to assess policy effects, the level of awareness observed among participants may partly reflect the influence of this broader policy context.

In addition to the retail venue, beneficiary identification and enrollment procedures may also influence the feasibility and scalability of HFSP. Automatic enrollment through existing social protection systems likely facilitated adoption by reducing administrative barriers and minimizing participant burden.The Bolsillo Saludable pilot program targeted households already enrolled in existing government assistance programs, similar to approaches used in initiatives in the United States [41]. This experience suggests that integrating HFSP within existing social protection systems may help reduce administrative barriers to participation and simplify beneficiary recruitment [21]. In addition, our findings highlighted the importance of allocating the full household benefit to the primary food purchaser, who were a woman in all participating households. Beneficiaries reported high program usability and described incorporating the subsidy into their routine food purchasing practices. This finding is consistent with previous research highlighting the importance of designing nutrition programs that avoid placing additional time burdens on women, who often bear a disproportionate share of food-related responsibilities due to gendered roles and the unequal distribution of household tasks [42,43].

The digital delivery system appears to have supported adoption and feasibility by simplifying benefit distribution and redemption, likely because smartphone and data/internet penetration in Chile is very high [44]. Online platforms have been used successfully in several countries, including Indonesia [45]. However, reliance on app-based delivery systems may create barriers for populations with lower digital literacy, limited smartphone access, or unstable internet connectivity, potentially excluding some older adults, rural populations, or more socially isolated households. Although smartphone ownership and internet connectivity are highly prevalent in urban Chile, these conditions may differ in rural or underserved settings. According to UNDP data [46], in 2023 Chile and Panama ranked above the OECD average in household internet connectivity, whereas Costa Rica, Brazil, Uruguay, Mexico, and Peru were above the Latin American regional average (75% of households). In contrast, internet coverage remains considerably lower in several other countries in the region and tends to be substantially lower in rural areas. In rural contexts, lack of internet access and technical expertise can hinder the implementation of fully digital platforms. A hybrid system combining tickets and electronic cards, as used in Mongolia [47] may represent a feasible alternative for improving accessibility and scalability across more diverse populations and implementation contexts.

Regarding the incorporation of nutrition education, the Bolsillo Saludable pilot did not include a nutrition education component. However, evidence suggests that integrating nutrition education into HFSP often enhances nutritional knowledge and encourages healthier food consumption across both high- and low-income settings [48]. Such components may also help reduce the gap between the financial benefit provided and the observed changes in household expenditure on FV by supporting households in prioritizing healthier food choices within constrained budgets. This may be particularly relevant for low-income households, which often face competing financial demands and must allocate limited resources across multiple essential needs, such as transportation, utilities, or medications. Incorporating a well-designed educational component into future iterations of the Bolsillo Saludable program could strengthen its potential to improve dietary diversity and reduce the consumption of UPF. However, as identified during the formative stage of the study, successful implementation would require careful attention to the design, timing, delivery format, and scheduling of educational activities to maximize participation and minimize additional burdens on participants. Integrating educational strategies into existing community or social protection platforms may represent a feasible approach for future scale-up.

### Feasibility challenges

The pilot faced several logistical challenges, such as vendors’ limited experience with administrative processes required for the payment system and the small number of participating stalls at the *Feria Libre*. These barriers highlight the need for continued support to strengthen the feasibility of implementation, particularly by assisting vendors with administrative and financial tasks such as invoicing, payment processing, and the adoption of digital tools. In addition, the limited number of participating stalls constrained beneficiaries’ purchasing options, reducing the variety of FV available and limiting common shopping strategies, such as comparing prices across stalls or distributing purchases across multiple *Ferias Libres*. Addressing these operational barriers will be important to improve program feasibility and support the potential scaling up of the intervention.

### Sustainability and scalability

Consistent with Proctor et al.’s sustainability outcome, long-term maintenance of the intervention will depend on the continued availability of financial, technological, and institutional resources. In the Chilean context, some administrative burdens may be reduced because the program was built upon pre-existing social protection registries and digital platforms already used for governmental benefit delivery. However, large-scale implementation would still require sustained investment in digital infrastructure, payment systems, data protection, and cross-sector coordination. Scaling up would also require strengthening vendor onboarding and support for invoicing and digital payment adoption. The growing use of electronic payments among *Feria* vendors and high smartphone penetration in urban Chile support the long-term feasibility of this model, though future evaluations should assess sustainability across more diverse territorial contexts.

### External validity and generalizability

The high levels of adoption and acceptability observed should be interpreted in light of the study population’s characteristics. Self-selection may have favored households with greater digital literacy and community engagement, given that beneficiaries were recruited through neighborhood councils and were already regular *Feria Libre* shoppers and participating vendors had prior experience with electronic payments. However, these characteristics are not uncommon among low-income urban populations in Chile, where smartphone ownership, internet connectivity, and electronic payment adoption among *Feria* vendors have expanded considerably in recent years [44,49]. Findings may therefore not be directly generalizable to populations with lower digital literacy or limited internet access, particularly in rural or socially isolated settings.

### Implications for future policies

The high levels of adoption and acceptability observed in this pilot, together with the reported changes in consumption patterns suggest that a FV subsidy may represent a promising strategy for improving access to healthier foods among low-income population in urban Chile. Beyond providing financial support, the Bolsillo Saludable experience highlights the potential value of combining economic incentives with food environment approaches that guide purchases toward healthier options while supporting local food systems.

Several implementation features of the Bolsillo Salusable program may be particularly relevant for future policy development. First, integrating healthy food subsidies within existing social protection systems may reduce administrative barriers, facilitate beneficiary identification and enrollment (primary FV purcharser at home), and improve program accessibility. Second, the use of traditional food markets such as *Ferias Libres* may offer an effective mechanism for reaching vulnerable populations while simultaneously supporting small-scale traders and strengthening local food systems. Given the widespread presence of traditional food markets across Latin America, this approach may have relevance beyond the Chilean context. The pilot also suggests that digital delivery platforms can facilitate benefit administration, monitoring, and redemption processes when adequate levels of connectivity and digital literacy are present. However, successful implementation at scale will require continued investment in technological infrastructure, vendor support, and administrative capacity. In particular, ensuring adequate infrastructure, support for vendors, expanding the number of participating stalls, and strengthening operational systems will be important to improve program feasibility and sustainability.

Future iterations of the program could also consider expanding eligible foods to include other nutrient-dense foods such as legumes, eggs, and fish, that could help meet a broader range of dietary needs. The results of this intervention also underscore the importance of integrating nutrition education strategies alongside food subsidies, which may help maximize the long-term replacement of UPFs with healthier options

Importantly, the formative research approach used to design this pilot program may represent a valuable strategy for adapting healthy food subsidy interventions to diverse local realities. Rather than replicating a fixed model, formative research can help identify context-specific barriers and facilitators related to food environments, social protection systems, retail structures, digital infrastructure, and beneficiary preferences. This approach may support the development of culturally appropriate and operationally feasible interventions across different territories and countries.

### Limitations

A primary limitation of the study is the small sample size and the short duration of the pilot program, which restricts the ability to assess the long-term effects on participants’ dietary behaviors and health outcomes. Additionally, the study was restricted to an urban setting while potential challenges could emerge in rural settings, specifically on available internet networks and digital literacy. Furthermore, the lack of a control group limits the capacity to establish direct causal relationships between the intervention and observed outcomes. Therefore, observed changes in expenditure, purchases, and dietary indicators cannot be interpreted as causal effects of the intervention. External factors, such as fluctuations in FV prices and seasonality, could have influenced spending patterns and purchases during the study period. However, price fluctuations were largely aligned with overall consumer price index changes for FV, which helps control for potential bias due to price changes.

## Conclusions

This feasibility pilot demonstrated high levels of adoption, acceptability, and appropriateness of the Bolsillo Saludable program, a smartphone app-based healthy food subsidy intervention implemented through Ferias Libres among low-income households in urban Chile. The findings suggest that combining economic incentives with healthy food restrictions and traditional food market delivery may represent a feasible and acceptable approach for improving access to FV while supporting local food systems.

The preliminary results suggest that the Bolsillo Saludable subsidy may support access to FV and may be associated with positive dietary changes among vulnerable populations; however, these findings should be interpreted cautiously given the pilot design and absence of a comparison group. To maximize its effectiveness, addressing logistical challenges and increasing the diversity of available products is essential. Future studies should explore the sustainability and long-term impacts of such interventions, as well as their scalability at a national level.

## Author contributions

The authors’ responsibilities were as follows—CC, SWN, LST, and IP: designed the research and methodology; IP and DMdO: conducted the research, analyzed data, wrote the manuscript; all authors: critically reviewed and edited the manuscript, have responsibility for the final content, and read and approved the final manuscript.

## Data availability

Data described in the manuscript, code book, and analytic code will be made available upon request pending (e.g., application and approval, payment, other).

## Declaration of Generative AI and AI-assisted technologies in the writing process

The authors declare that no generative AI or AI-assisted technologies were used in the writing of this manuscript.

## Funding

This work was supported by Bloomberg Philanthropies, Healthy Food Policy Program, grant number 5128637. Additional support was received by Fondecyt Regular (CC, #1240833) and ANID-Subdirección de Capital Humano/Doctorado Nacional/2024-21240462 (DMdO). Supporting sources had no involvement in the study design, data collection, analyses, interpretation or publication.

## Conflict of interest

The authors report no conflicts of interest.
