# Supplementary material for: Adoption and Acceptability of a Fruit and Vegetable Subsidy in Low-Income Households in Urban Chile: The Bolsillo Saludable Pilot Feasibility Study
Source: Curr Dev Nutr. 2026 Jun 13;10(7):109394. doi: 10.1016/j.cdnut.2026.109394 (PMC13355171; doi:10.1016/j.cdnut.2026.109394)
Supplement: multimedia component 1 [file mmc1.docx]

**Adoption and acceptability of a Fruit and Vegetable Subsidy in Low-Income Households in urban Chile: the Bolsillo Saludable pilot feasibility study**

Pemjean, Isabel

**Supplementary Figure 1**: Total and average expenditure by day.


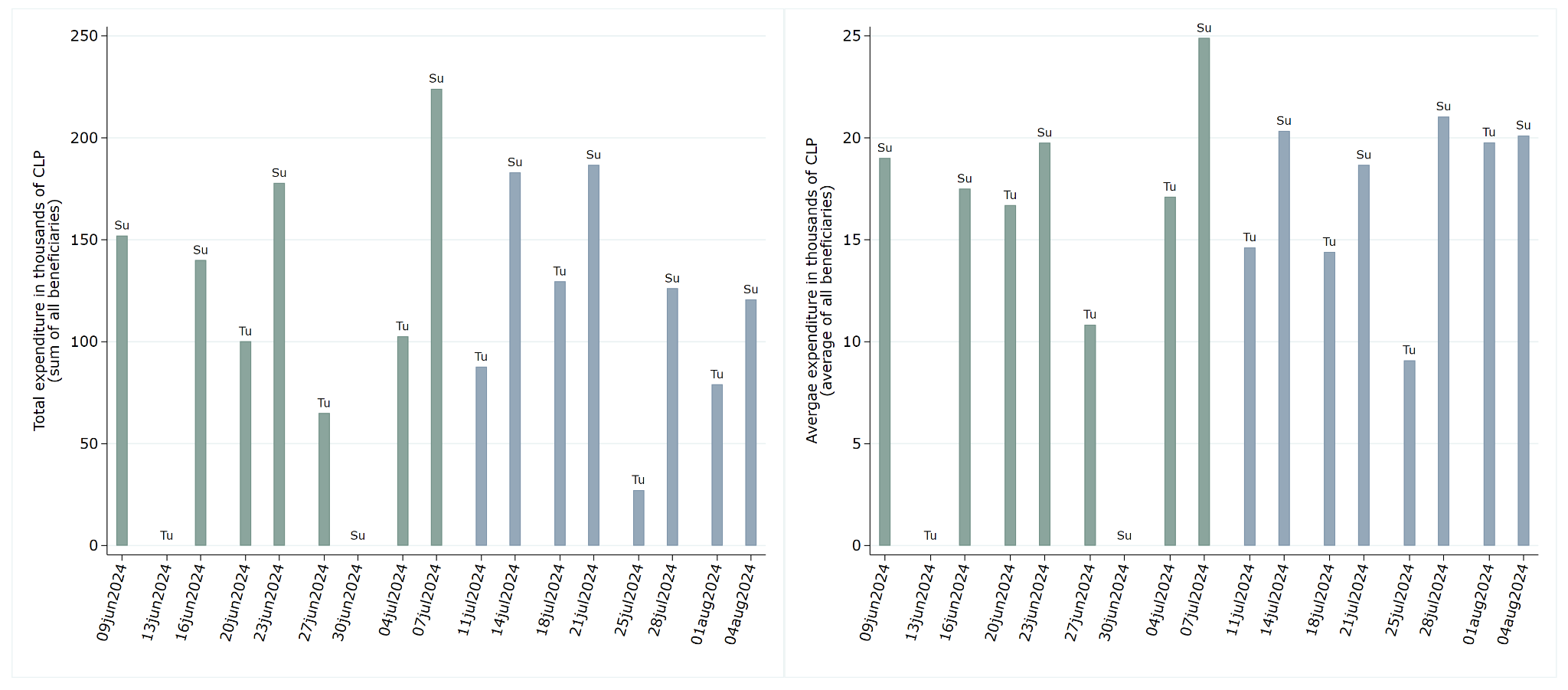


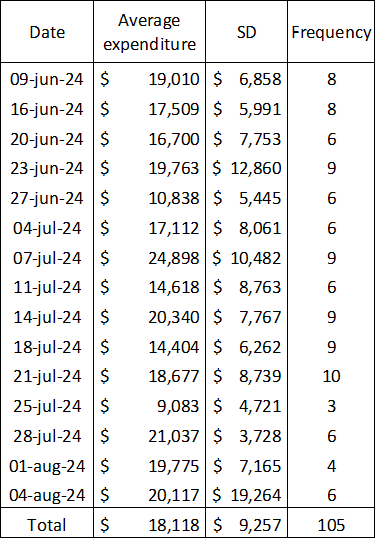


Supplementary figure 1 1 shows the amount of money spent and the average amount spent by beneficiaries who shopped on each day of the pilot. Since the Ferias Libres are open on Thursdays (Tu) and Sundays (Su), we can see that Sundays were the days with the highest amount of money spent.

**Supplementary figure 2:** Number of buyers and sales share per market stall.


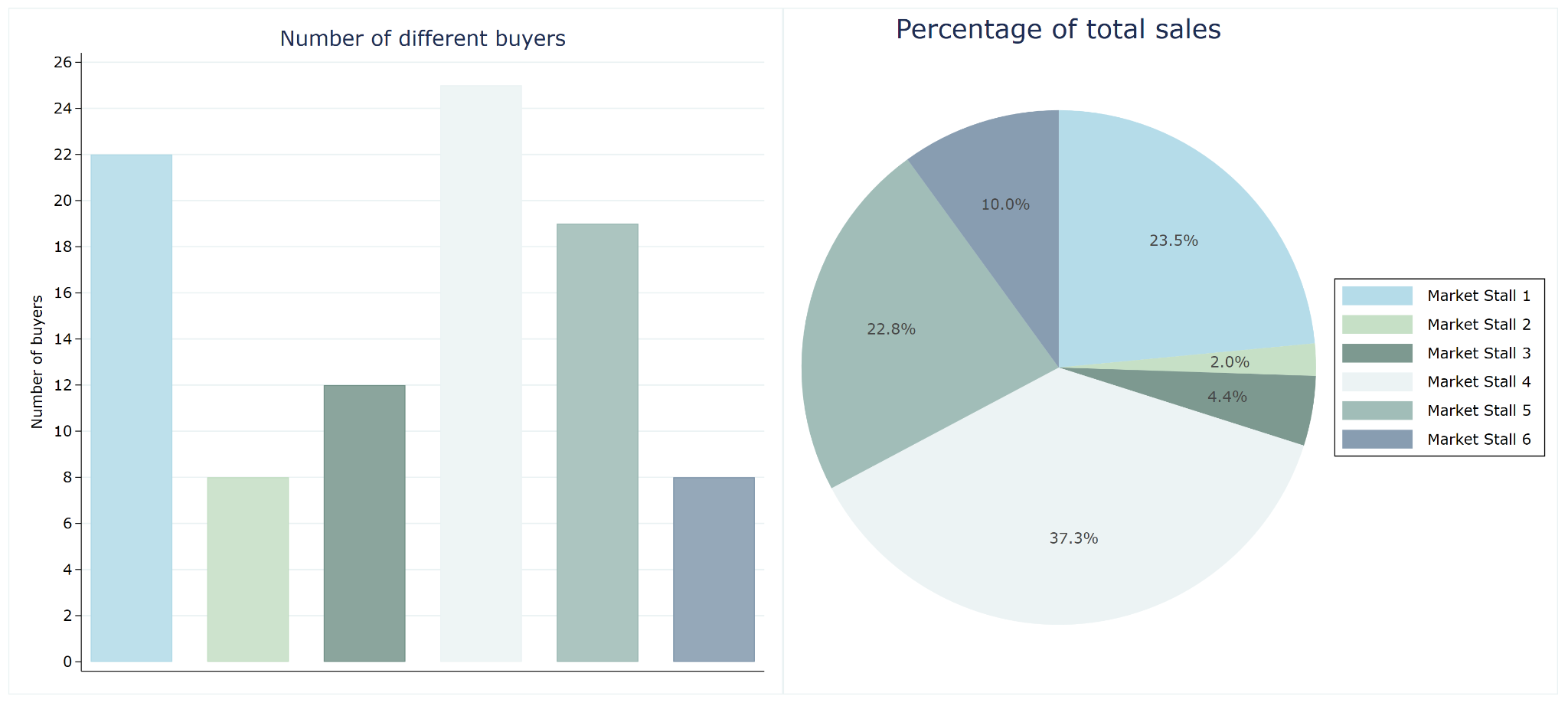


**Supplementary figure 3:** Fruits and vegetables expenditure, purchases in CLP.


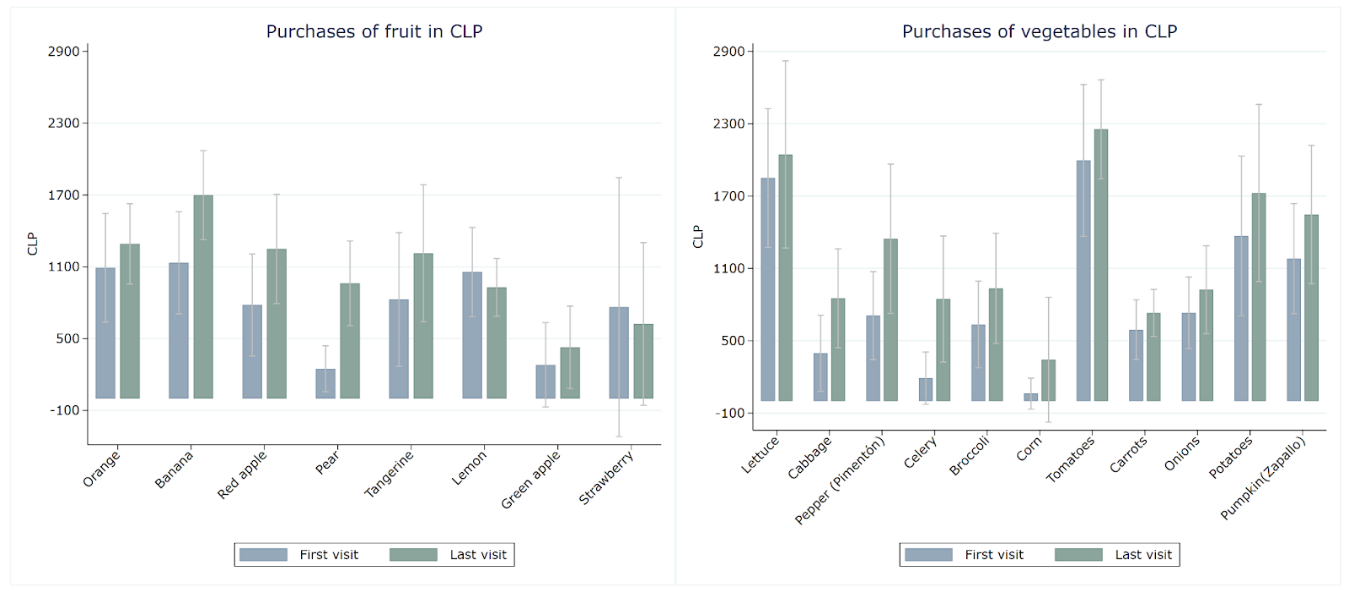


Supplementary figure 3 shows the average expenditure on each fruit and vegetable reported by the beneficiaries during the first and last visit.
